# Biobanking for Translational Diabetes Research in India

**DOI:** 10.1089/biores.2019.0052

**Published:** 2020-08-19

**Authors:** Charitha Gangadharan, Soniya Wills, Rajani Kanth Vangala, Alben Sigamani

**Affiliations:** ^1^ Department of Clinical Research, Narayana Hrudayalaya Foundations, Bommasandra, Bangalore, India.; ^2^ Institute for Applied Research and innovation (InARI), Chikkalasandra, Bangalore, India.

**Keywords:** biological specimen bank, certification, diabetes, ethics and legal issue, India

## Abstract

India is declared as the diabetic capital of the world. Clinically well-annotated blood samples will advance diabetes research for better diagnostic and treatment methods. Building a disease-specific biobank with high-quality peripheral blood mononuclear cells (PBMCs) and clinical follow-up data system will serve as a good platform for clinical research in diabetes. Processing and storage of high-quality biospecimen for translational research in diabetes demand the implementation of good clinical laboratory practices. “Certification or accreditation programs” that improve biorepository processes and frameworks are lacking in Indian context. To sustain and translate the research into clinical practice, good governance of the biobank and financial resources is required. For ethical issues related to health needs of the people and participants in the research, issues related to research process, translational research, and commercialization, data sharing should be addressed. For India to be an innovation and sustainable country Indian government is supporting translational research facilities, including biobanks. India has developed biobanks for various diseases; however, diabetes-specific research biorepository is lacking. Given the dangers of diabetic burden, India should set up a diabetes disease-specific repository learning from the global organizations and customize to the needs of Indian context. It is important to have private agencies get involved to develop biobanks and future research as there are commercial goals to translate research into practice. New technologies of specimen storing and preservation, data management, and data sharing should be adopted for developing cost-effective long-standing disease-specific population biobank in India.

## Introduction

Diabetes is a major, common, costly, yet preventable disease with early detection, lifestyle modifications, and therapy. However, the global prevalence of diabetes is increasing. Forty-nine percent of the world's diabetes burden is represented by India, with an estimated 72 million cases in 2017. The proportion of undiagnosed diabetes is also high in India, estimated to be 58%.^1^ Diabetes and its complications are known to contribute significantly to mortality, costs, and poor quality of life. Evidence suggests that the mortality rate is three times higher in diabetic individuals compared to those without diabetes.^2^ Diabetes management is still a clinical challenge; identifying new biomarkers that have the potential to predict the staging and disease progression and monitoring therapeutic intervention will aid clinicians in better management of the disease.

Peripheral blood samples are frequently used in diabetes research for developing better diagnostic and treatment regimes. Clinically well-annotated peripheral blood samples are used in developing new treatment methods,^3^ assessing diabetes risk and predicting associated complications at an early stage,^4,5^ identification of high-risk population for disease occurrence and progression,^6,7^ etc. Access to high-quality well-characterized biospecimens, including PBMCs, to bring new therapies to patients in need will accelerate diabetes research.

India is declared as the diabetic capital of the world, and increasing incidences of this disease especially in the younger population is a serious concern. Emphasis on better management of the disease by developing more sensitive methods of detection and effective regimen of treatment is the need of the hour. The disease is characterized by abnormal levels of glucose resulting from the defects in insulin secretion, insulin action, or both. Much of the research on pathophysiology of the disease and its complications are done on animal models. At present, specialized biobanks to validate discoveries made in animal studies does not exist in India. So, developing and building a disease-specific biobank for diabetes can facilitate basic and clinical research and will allow us to better understand chronic disease like diabetes. Carefully designed and established biobank with large cohort of patients diagnosed with diabetes subtypes, covering different geographical areas, will serve as a valuable resource to study risk factors, including molecular biomarkers for disease progression and complications.^8^

## Types of Biobanks in Diabetes Research

A biobank is a type of biorepository that collects, processes, preserves, and stores high-quality biospecimen (of microbes, plants, animals, including humans) and its associated data for use in research and clinical care. The number of biobanks has increased considerably across the globe, however lacking in low- and middle-income countries. Biobanking started with small university-based repositories that were largely developed for research needs in specific projects. With the increasing demand for human biospecimen in academic and pharma industry biorepositories evolved as institutional and government supported repositories, commercial (for profit) biorepositories, population-based biobanks, and most recently virtual biobanks.^9^ Depending on the type of sample stored specialized biobanks, including disease centric, genome biobank (genetic or DNA/RNA), and tissue/multiple specimen type biobanks were developed.

Population-based biobanks are huge repositories created to collect, analyze, and store phenotypic and genotypic information on representative samples of their source population. These repositories are developed by countries, including United Kingdom, Denmark, Canada, South Korea, Japan, Singapore, United States, and Iceland. These types of repositories require widespread collaborations for funding, governance, and maintenance. Population-based biobanks are best suited to study rare diseases and genetic variants with small effects.^10^

Hospital-based biobanks or single institutional biobanks are developed by investigators for their research projects and include a smaller collection of samples or samples from multiple studies with common storage and governance.^11^ When the biospecimen and the data integrated are dispersed in biobanks in multiple locations, virtual biobanks designed to connect biobanks through specialized software or web portals are established for broad-based sharing of research resources.^12^ Disease-specific biobanks are generally developed in hospitals for epidemiological studies and focus on amassing large population of samples from persons with specific disease conditions such as AIDS, cancer, diabetes, and so on.

In the era of precision medicine integrating proteomics, genomics, and personal medicine well-curated biobanks will contribute to research and discovery. A variety of biobanks are established to accelerate translational research in response to changing needs of technology, investigators, and regulatory policies. However, establishing a specialized biobank in developing countries like India would meet the translational research needs and effective use of biospecimen for “omics” research. Evidence from breast cancer research suggests that the contribution of India in breast cancer research is increasing and an increasing trend in international collaborative studies with top countries during the past decade.^13^ The knowledge of breast cancer and high-quality translational research in breast cancer was possible in India because of small-scale high-quality biobanks developed by institutional biobanks. The repository model of breast cancer in India might aid in defining successful therapy in diabetes as well.

## Requirements to Build a Biospecimen Repository

A diabetes disease-specific biobank will serve as a resource for investigating many clinically relevant research questions. A repository with complete and accurate clinical data linked to biospecimen only will have the potential of future scientific utility. Hence good laboratory practices must be adopted when acquiring the specimen, as well as adequate resources to build, store, and sustain the collection. Several working models of biospecimen repository exist, and specimen collection can be done retrospectively or prospectively according to study needs.^14^

### Infrastructure, facilities, and equipment

The International Society for Biological and Environmental Repositories (ISBER) has set in guidelines to design biorepositories. The facility must ensure safekeeping of the material stores, support the equipment use, and provide safe and effective working environment for the repository personnel. Adequate freezer space, maintenance of the equipment for sample processing and storage, power supply and adequate backup, and temperature monitoring should be provided round the clock. Storage space with enough ventilation and air conditioning to accommodate ultralow temperature (Vapor phase of liquid nitrogen or −80 freezers) is needed to store different types of specimen according to the study needs.^15^

IT infrastructure to track and monitor biospecimen location and associated data is also necessary. A robust electronic inventory system to capture collection, processing, shipping, and storage information associated with individual vial is essential.

### Budget

For developing a quality biospecimen collection, financial resources are required. The budget can be planned out in phases (start-up phase, operational phase, and cost recovery phase). The budget should include people, facilities, and time needed to collect, annotate, process, monitor, and preserve the specimen. In case of collaborative studies “study closeout” activities and physical transfer of the material should be included in the budget.^15^

### Resource personnel

Selection of personnel with expertise in designing, building, and maintaining biospecimen collection is ideal. The personnel should be trained in standard operating procedures (SOPs) for sample and data collection, preservation, and inventory. They should be aware of the consenting process and regulations for proper transport of biospecimen. Experience in sample processing and preservation techniques, collation, building and implementing quality assurance and control, and preparing electronic files and link to clinical demographic data are also necessary.^15^

### Certification of biobank

Apart from infrastructure, budget, and resource personnel, core aspect of biobanking is the biospecimen quality and good governance. Good Clinical Laboratory Practice (GCLP) implementation provides assurance that the collection processing and storing of the samples are planned, performed, monitored, recorded, and reported in a reliable and consistent manner, which in turn increases the credibility of the biobank in terms of accuracy, integrity, and traceability of the generated data and the specimen stored. Quality assurance and quality control review the research process to adhere to SOPs to determine accuracy of research records. External quality assurance programs or audits aid the laboratories to ensure that the data generated are accurate and clinically appropriate. In addition, it provides the sponsors or collaborators that the data generated are of high quality.^16^

Accreditation or certification along with educational programs for biobanking focusing on rationale and the standards set is an adaptable and flexible mechanism to address both quality assurance and governance. A certification program developed by Canadian tumour repository network (CTRNet) based upon the education of all repository personnel in multiple areas of biorepository operations (SOPs, bioethical and regulatory issues) is now available internationally to biobanks.^17^ Biobank certification programs at national level in United States are conducted by College of American Pathology (CAP), which utilizes checklist to identify and solve problems as to quality of laboratory operations.^18^ The chief regulatory body for repositories in India is Indian Council of Medical Research (ICMR). ICMR has set guidelines for GCLP; however, there is lack of regulatory agencies for certification of biorepositories in Indian context.

It is highly recommended that “certification programs” will improve capacity and quality of the biospecimen through standardization and improve the biobanking processes and frameworks. In addition, registration of clinical trials when it begins aids in making the information publicly available and benefits a variety of people; however, registering the biorepository to a regulatory agency does not exist. The process of registration of biobank will legally authorize its existence and improve public trust toward biomedical research in India.

### Ethical and legal issues of a biobank

The biobanks deal with human samples, invading individual autonomy entailing significant ethical obligations. The ethical questions, such as the what good purpose or health benefit of the population is addressed and met by the proposed research, the competence of the participant in getting involved in the study and the implications on participation, the ownership of the sample, and the results of the research, are important to be addressed. The major ethical issues with biorepositories are (1) principles for responsible custodianship, (2) informed consent, (3) privacy and confidentiality protection of donors, (4) access to biospecimens and data, and (5) intellectual property and resource sharing.^19^

As per the ethical model of custodianship, the caretaker of the biobank has control over the biospecimens and avoids any conflicts between the stakeholders.^20^ They are obliged to ensure that the biospecimens are used to facilitate valuable biomedical research. Every biobank should have teams for proper governance, management, resource support, and institutional support.

The informed consent and privacy in biorepositories are largely dependent on the concept of autonomy—that patients can participate on voluntary basis and can withdraw from the study at their own will. As per the regulatory guidelines special care should be taken to deidentify the participant information. Maintaining the privacy of the specimen/data is of utmost importance to enhance the trust of patient population in the institution and increasing participation in biorepository building. Investigators and the biobank in charge should decide on the minimum identifiers that will be maintained in the specimen and the data they use from the repository. A broad consent, with consent for future research with a potential disclosure of results or for further additional information or samples, is ethically recommended for biobank research.

Research institutions and hospitals are involved in multilevel collaborative studies where more samples and data are being shared. Data are also shared for commercial gain with third parties as the health data are being captured outside traditional health care settings. The protection of privacy of the contributors of the biospecimens is a major concern in the biorepositories. The confidentiality of the data collected from these repositories should also be maintained by ensuring best ethical standards. Biorepositories of high standards like the “The National Cancer Institute” ascertain well-defined guidelines for access to biorepositories. Existence of data access committee responsible for avoiding redundant studies and ensuring the best use of the biospecimens is recommended for all biorepositories. The information and data derived from these repositories are of immense commercial value. The policies for intellectual property (IP) vary from institution to institution; hence, standardization of these policies is mandatory. The *NCI Best Practices for Biospecimen Resources* recommends timely sharing of the data and information generated from the biorepositories.^21^

Significant ethical obligations are associated with the use of data and biospecimen; issues like who owns the stored biospecimen as substantial property, concerns of the participants and maintaining autonomy, commercial gains, and proper utilization of the contributed biospecimen are essential to getting to the kinds of advances in science to promote the health care that we need. However, in India we are lacking a well-annotated biospecimen bank to provide the specimen and associated data for research on diabetes.

## Biorepositories in India

India is a country with a diverse ethnic population and very high burden of communicable and noncommunicable diseases. Therefore, the Indian population is an important resource for research material. Building India as an Innovation nation is a national priority, and the Indian government has supported development of translational research facility, including biobanks.

India has a commercial biobank for its credit established as a joint venture between Apollo hospitals and Saarum innovations. In addition to the challenges faced by lack of awareness, paucity of research budgets, infrastructure, and social and ethical barriers, India has established biobanks (Table 1). However, India lacks a disease-specific population biobank for diabetes. With strong support from the Indian government translational cancer research has progressed with establishment of regional biobanks and linking them with overarching national cancer biobank. The establishment of national cancer grid has led to connecting the existing and proposed cancer centers and biobank for high quality and uniform diagnosis and treatment protocol across the country. India with more than half of the global diabetes burden, absence of a major diabetes-specific research biorepository is a major concern.

**Table 1.** Examples of the Established Biobanks in India

Biobank/institute	Location	Website	Disease
NCTB	Chennai, Tamil Nadu	https://nctb.iitm.ac.in/about/index.html	Cancer
NLDB	New Delhi	www.nldb.in/	Liver diseases
Rajiv Gandhi Cancer Institute and Research Centre	New Delhi	www.rgcirc.org/research/biorepository/	Cancer
NIMHANS	Bangalore, Karnataka	http://thenimhansbrainbank.in/	Brain disorders
TiMBR	Kolkata, West Bengal	www.web.tmckolkata.com/biobank/index.html	Cancer
National Cancer Institute-All India Institute of Medical Sciences	Jhajjar, Haryana	http://nciindia.aiims.edu/en/node/16	Cancer
Sapien Biosciences	Hyderabad, Telangana	http://sapienbio.co.in/biobank/	Commercial biobank
National repositories for cell lines/hybridomas NCCS	Pune, Maharashtra	www.nccs.res.in/index.php/TeamsNCCS/Repositories	Cell lines, microbial resources, iPSCs
ADBS	Bangalore, Karnataka	https://www.ncbs.res.in/adbs/bio-repository	Brain disorders using stem cells

ADBS, Accelerator Program for Discovery in Brain Disorders Using Stem Cells; NCTB, National Cancer Tissue Biobank; NIMHANS, National Institute of Mental Health and Neurosciences; NLDB, National Liver Disease Biobank; TiMBR, The Tata Medical Centre Biorepository.

## Existing Diabetes Biorepositories

Specialized diabetes biobank is an invaluable resource, and many international biobanking organizations have already been established. These organizations have developed guidelines to address the technical, legal, and ethical issues relevant to biorepositories, which clearly indicate that the domain of biobanking is a scientific and professional entity. Table 2 below is some of the most well-known specialized biobanks for diabetes.

**Table 2.** Examples of Existing and Promising International Specialized Biobanks for Diabetes

Sl. no.	Name of the repository	Country	Year	Condition	Biospecimen	Sample Size	Reference
1	National Institute of diabetes and digestive and kidney diseases	United States	2002	Diabetes, kidney disease, liver disease, and inflammatory bowel disease	DNA, Plasma, serum, urine, RNA, PBMC, stool samples	over 5000000	^22^
2	nPOD	Florida	2007	Type 1 diabetes	Pancreas and related tissues (spleen, lymph node, pancreatic lymph node, peripheral blood, thymus, and bone marrow) from cadaveric organ donors	160	^23^
3	UK Biobank	London	2006	Cancer, stroke, diabetes, heart disease, eye disorders, depression, dementia, and arthritis	Blood, saliva, and urine	500000	^24^
4	The Danish Centre for Strategic Research in Type 2 Diabetes, DD2	Denmark	2010	Type 2 diabetes	Whole blood, DNA, plasma, and urine	50000	^25,26^
5	Qatar Biobank	Qatar	2012	Cardiovascular disease, obesity, diabetes, and cancer	Blood, urine, saliva	60000	^27^
6	China Kadoorie biobank	China	2004	Heart attack, stroke, diabetes, and cancer	Blood	510000	^28^
7	Diabetes Pearl	Netherlands	2002	Type 2 diabetes	storage of urine and blood samples and isolated DNA	7000	^29^

PBMC, peripheral blood mononuclear cell.

## Future Perspectives

One of the major challenges for long-term sustenance faced by biobanks is financial support. Financial support from multiple sources like the commercialization of research results or derived products, cost recovery related to access to samples, funding from private for-profit entities such as biotech companies, and funding through government institutions or agencies might ensure long-term financial viability.^30^ The total cost of accruing, processing, and managing samples can be shared among the users who are using the samples for research. Other cost reduction strategies such as reducing the investigations that do not benefit to patient's health care needs and avoiding redundancy by sharing samples and associated data will be beneficial. The commercialization of research results or derived products may be challenging to rely as a steady source of income as biobanks cannot claim any ownership of the intellectual property rights that are developed by third parties by accessing the samples and data from the biobank. Studies and surveys suggest that the involvement of a commercial entity in public biobanking activities may affect patient's willingness to take part in and consent to research. One of the solutions to the problem of funding is to get integrated into the health care systems that use longitudinal samples from individuals as a part of their patient care. Consolidating and scaling up can reduce the unit cost, sharing samples among institutions, and impending global funding mechanisms are strategies to improve long-term financial sustainability.^31^

The material stored in the biobank is a treasure as it can be utilized in personalized medicine tackling aspects of prevention, diagnosis, treatment, and monitoring specific characteristics of an individual patient using omics technologies. Standardized protocols and SOPs for the collection and storing of the biospecimen were used to ensure high-quality biospecimen samples. Long term storage of biospecimen continuously demands capital cost and recurring cost. However, preservation science has progressed considerably in preserving therapeutic proteins and drugs. Integrating the preservation science from other fields to the biospecimen preservation to improve quality and cost is an unmet need.

New technologies can be developed to reduce time and labor involved in processing of biospecimen and improve the quality.^32^ Technologies have improved in data collection as well, as there are potentials to enter the data from multiple digital sources. Leveraging the data sources and integrating them with the biorepository can increase the knowledge base and utility of sample.^33^

Biorepositories do not generate profits; however, sustaining itself with quality and cost is challenging. Models of international and interdisciplinary collaboration and sample sharing and better preserving technologies may address some of the challenges in long term sustainability. Disseminating experimental lessons learned and scientific data across the scientific community and society might be a model where people will willingly come forward to donate their samples and personal information. Important to have private agencies permitted to create such biobanks for future research. Databases located only within regional and academic institutes have a drawback—there is no commercial goal to translate research into action. There is no value in creating large amount of data that is converted into publications without a go-to-market drive to transform the lives of people suffering from the disease. Hence, the goals of any database must be to translate knowledge to practice.

In conclusion, reduction of health care cost is only possible with development of long-standing well-characterized biobanks with constantly developing new approaches and ideas.

## Abbreviations Used

ADBS: Accelerator Program for Discovery in Brain Disorders Using Stem Cells
CAP: College of American Pathology
CTRNet: Canadian Tumour Repository Network
GCLP: Good Clinical Laboratory Practice
ICMR: Indian Council of Medical Research
ISBER: International Society for Biological and Environmental Repositories
NCTB: National Cancer Tissue Biobank
NIMHANS: National Institute of Mental Health and Neurosciences
NLDB: National Liver Disease Biobank
PBMC: peripheral blood mononuclear cell
SOP: standard operating procedure
TiMBR: The Tata Medical Centre Biorepository
